# Dog attacks on livestock: insights from Swedish news articles and experiences of farmers and inspectors

**DOI:** 10.3389/fvets.2025.1629966

**Published:** 2025-10-31

**Authors:** Sirkku Sarenbo, Marie Doane

**Affiliations:** Department of Biology and Environment, Faculty of Health and Life Sciences, University of Linnaeus, Kalmar, Sweden

**Keywords:** dog attack, livestock protection, wild predators, public awareness, supervision of dogs

## Abstract

**Introduction and purpose:**

Wild carnivore predation on Swedish livestock has been meticulously recorded, but dog attacks on livestock tend to be overlooked. This study aimed to map the nature of dog attacks on livestock.

**Material and methods:**

Data was collected from Swedish news media articles, Rovbase records in the region Västra Götaland, and electronic online surveys of farmers and official inspectors. In addition to descriptive statistics, monthly indices of dog attacks on livestock were calculated using data from Rovbase and news media articles.

**Results:**

Half of the inspectors had inspected livestock attacked by dogs. Dogs accounted for 3.8% of all predator attacks inspected in Västra Götaland 2004–2024. Most dog attacks occurred during summer and fall. Sheep were most often subjected to dog attack, followed by horses. Sheep were most often attacked by dogs on pastures, hens in their enclosed barnyard, and horses when ridden or driven. The attacking dogs were most often unknown, loose, or unsupervised.

**Discussion:**

Dog attacks impact both animal welfare and societal interests. The farmers expressed emotional distress after the dog attacks. Misidentification can worsen wolf-related conflicts and misdirect public funds, while livestock owners may face economic losses despite the dog owner’s sole responsibility. Further research is needed to assess dog attacks on livestock nationwide. However, targeted measures such as predator-deterrent fencing, prolonged mandatory leashing of dogs, and enhanced monitoring of grazing livestock during hunting seasons could already be implemented.

## Introduction

Our relationship with dogs (*Canis lupus domesticus*) is complex, and how dogs are perceived may vary depending on cultural, geographical, historical, and religious contexts (1). There were 1.156 million registered dogs and 0.835 million registered dog owners in Sweden as of 31^st^ December 2024 (2). These dogs are primarily used for companionship or hunting, but they also play a role in agriculture, the tourism industry, healthcare, military, police, and Swedish Customs Service. To date, there are no known packs of feral dogs in Sweden (3).

Dog attacks on livestock have been documented in several studies; however, attacks carried out by dogs are still sometimes attributed to wolves and other wild predators (4, 5). The attacks have been documented across the European continent, from northern to southern regions (6). However, accurate estimations of the total number of dog attacks remain challenging due to the absence of a centralized database for reporting such events. Moreover, the topic has received limited attention within the scientific literature. However, dogs pose a threat to livestock and should be considered in mitigation plans when assessing the impact of predation by different predator species, including wild ones. Dogs can act both alone and in groups, and they can be escaped pet dogs or feral dogs that form packs (5, 7). Dogs behave differently than wild predators do. They generally run up the side of the prey animal and bite many times wherever they can, thighs, legs, back, neck, usually low down, while wild predators are significantly more skilled and cause fewer bites on specific attack areas, usually higher than dog bites (8–10). Bite wounds may appear minor on the surface, but they can cause significant internal damage and infections in the underlying tissues that are not immediately visible. As a result, bite injuries almost always require prompt antibiotic treatment—or, in some cases, euthanasia—to ensure the animal’s welfare (8). Sweden is so far free from rabies, but it has been shown that imported dogs from risk areas lack protection against rabies, which may be due to the vaccination not being carried out correctly (11).

The relationship between rural communities and native carnivores, such as wolves (*Canis lupus*), is complex and often contentious. While wolves play a key ecological role, their presence poses significant challenges to rural livelihoods, especially in livestock farming. Notably, their ecological importance tends to be more widely recognized by urban societies, which can lead to differing perspectives and priorities (12). Recently, debates have intensified across Europe and the EU regarding the appropriate presence and population size of the grey wolf (*Canis lupus*) (13–16). Political interests can intensify tensions by exaggerating the perceived impact of large carnivores (14), increasing the potential for conflict with livestock producers.

A Swedish legislative inquiry into competitive livestock production practices that ensure good animal welfare is currently open to consultation. During this consultation, the presence of wolves was cited as a factor deterring animal producers from expanding their operations (17). However, the involvement of dogs in the predation of sheep and other livestock has not been documented in Sweden as it has in Italy (18–21), Spain (22), Poland (7) and Estonia (5), probably obscuring a more complex predation problem that need to be addressed before promoting the expansion of livestock production.

Here, we need to note that the term “predation” refers to the condition in which an animal, a predator, kills and wholly or partially consumes another animal (23). Feral dogs have been shown to hunt and consume their prey, which can consist of wild and domesticated animals (24), while domestic dogs kept by humans as pets or for other purposes rarely consume their prey after hunting and killing them (8–10). Therefore, we used the term “attack” instead of “predation.”

There is strict liability for Swedish dog owners or keepers, which means that damage caused by a dog shall be compensated by its owner or keeper, even if he or she did not cause damage [Section 19 Swedish Act (2007:1150) on the supervision of dogs and cats]. The owner or keeper is entitled to recover any damage paid by the owner from the person who caused the damage. Section 16 states that during the period from March 1^st^ to August 20^th^, dogs must be kept under such supervision that they are prevented from running loose in areas with wildlife. During the rest of the year, dogs must be kept under the supervision that they are prevented from chasing or pursuing wildlife when they are not used for hunting. Exceptions from Section 16 are provided in Sections 16–19 of the Hunting ordinance (1987:905): Hunting dogs that are suitable for their respective hunting purposes may be used by the hunting rights holder or by another person with the hunting rights holder’s consent during permitted hunting and hunting training. Additionally, special provisions regarding the supervision of dogs are found in the Reindeer Husbandry act (1971:437). There are also regulations aimed at protecting livestock. If a dog is found loose in an area where livestock are present, and if the dog cannot be captured and it is necessary to prevent harm to the animals, the dog may be killed by a person who owns or cares for the animals. The person who killed the dog is required to report this to the police as soon as possible [Section 14 Act (2007:1150) on the supervision of dogs and cats].

The Swedish Wildlife Damage Center (VSC) provides annual statistics on the damage caused by large grazing birds and large predators such as gray wolf (*Canis lupus*), lynx (*Lynx lynx*), brown bear (*Ursus arctos*), golden eagle (*Aquila chrysaetos*), and wolverine (*Gulo gulo*) (25). Dogs injured or killed by large carnivores are included in the VSC’s statistics and scientific studies (13) but cases where dogs are suspected to lie behind injuries or kill livestock are not systematically tracked. We find it important that wild predators are not held responsible for injuries caused by domestic dogs, and vice versa. It is equally important not to exaggerate the impact of wild predators on livestock losses. The role and number of dogs involved in attacks on livestock in Sweden are currently unknown and should be investigated to ensure accurate and adequate mitigation efforts for attacks caused by domesticated dogs. In this study, information gathered from available sources was used to characterize the problem of dogs attacking, injuring, and killing livestock.

The aim of this study was to map the extent and nature of dog attacks on livestock in Sweden using four different information sources. Using multiple sources to study a new area increases the credibility of the results and reduces the risk of error compared to relying on a single source. It also provides a broader perspective and thus a more nuanced understanding of the issue and helps to confirm any emerging trends or patterns. For example, farmers offer first-hand accounts of attacks on their livestock, describing in their own words how both they and their animals were affected. The County Administrative Board, as an official authority, provides an institutional perspective on these incidents. Meanwhile, media coverage serves as a complementary source, highlighting the social relevance of the issue and illustrating how it is reported over time, potentially revealing broader trends. The research questions are as follows:

- In what situations do dogs attack livestock?
- What characteristics do dog attacks have, and which livestock species are the targets of the attacks?
- Do the attacks occur by chance, or is there a discernible pattern? Are there similarities with studies from other countries?
- What consequences did the attack have on animals subjected to dog attacks?
- What are the possible societal consequences of dog attacks on livestock?

These results could serve as a foundation for larger-scale studies, suggest mitigation strategies, and support development of official statistics on dog attacks on livestock and horses.

## Materials and methods

Information about dog attacks on livestock species alpaca (*Vicugna pacos*), cattle (*Bos taurus*), goat (*Capra hircus*), horse (*Equus caballus*), pig (*Sus scrofa*), poultry (*Gallus gallus, Meleagris gallopavo, Anser anser f. domesticus, Anas platyrhynchos*), sheep (*Ovis aries*) was collected from four sources: Swedish news media articles, an online electronic survey to animal keepers, an online electronic survey to county administrative board inspectors who inspect predator-infested domestic animals, and inspection records from the database “Rovbase.” The study was conducted between October 2023 and January 2025. All data were processed using Microsoft Excel (Microsoft 365), which was also employed to produce descriptive statistics and visual representations.

### Swedish news media articles

Swedish news media articles were searched using three sources: Google, Media Archive, and Swedish Newspapers. The search was conducted using the term “dog attack” combined with different types of livestock. Google search generated varying numbers of articles and other media. To limit the scope, only the first 100 results were included for each combination of search words. The Media Archive is Nordic’s largest media database. It includes all major daily newspapers and most regional newspapers as well as radio and television (26).

Swedish Newspapers comprise a collection at the Swedish National Library, including almost all newspapers published in Sweden since 1645 (27). It allows browsing and free text search of Swedish newspapers (*N =* 1,470) from the 17^th^ century onwards. The following information was collected from the articles: the attacking dog (breed or type, number of dogs, owner known or unknown, owner present or absent during the attack), the victim (species, number of animals, consequences of the attack), date and time of the year, and circumstances during the attack.

### Surveys for farmers and inspectors

Our study do not include any information that can identify a person or an animal. Separate surveys for farmers and inspectors were created using Artologik® Survey & Report software. The written informed consent process is as follows. Both surveys contained an introduction letter in which the research project, project leader, and aim of the project were presented. They were also informed that answering the questions in the survey was voluntary and that no personal data were collected. It was possible to answer only once from an IP address. Certain questions were mandatory to answer in order to proceed with or complete the survey (see Appendices 1 and 2). It was possible to cancel participation in the survey at any time; however, but a submitted reply could not be withdrawn.

The introduction letter of the survey for farmers explained that “dog attack” referred to an event where one or more animals were disturbed/molested, injured or killed by one or more dogs (this did not include, for example, intentional work or herding of animals with dogs). Additionally, if several dog attacks have affected the farmers’ animals, the response should be based on the attack that the farmer judged to be the most serious.

Both open and closed questions were included (Appendix 1). The survey was sent to the Swedish Sheep Breeding Association, who published it in a weekly letter. It was also sent to Swedish alpaca owners through social media. The comment fields in several questions offered an opportunity to provide further comments if the event was not covered by the options. The survey was open from 8^th^ July to 15^th^ September 15, 2024.

The survey for inspectors (Appendix 2) was directed at the inspection personnel on county administrative boards. A link to the survey was sent by email to the 21 county administrative boards of Sweden and to email addresses listed as inspection personnel at the Swedish Wildlife Damage Centre. The survey was open between 13^th^ and 31^st^ of January 2025.

### Rovbase records from Västra Götaland 2001–2024

We chose to investigate the occurrence of dog attacks in Västra Götaland because this county accounted for approximately one-third of all predator attacks that received government compensation in 2023 (25). Rovbase is a database that records the inventories of brown bears (*Ursus arctos*), wolverines (*Gulo gulo*), eurasian lynxes (*Lynx lynx*), gray wolves (*Canis lupus*), and golden eagles (*Aquila chrysaetos*) in Sweden and Norway. It also documents the damage these animals cause to domestic animals, as well as details of hunting and other causes of mortality (28). When a suspected predator attack on livestock is reported to the county administrative board, an inspector inspects the carcass, injuries and surrounding clues. The report is the basis for compensation that the Swedish state pays to the livestock owner for the damage caused by a wild predator. The inspectors recorded the type of predator, physical damage to the livestock or dog, and other relevant facts. However, there were no statistical records or official monitoring of dog attacks. Some inspectors do note in the report and enter it into Rovbase if they believe that the attack was performed by a dog instead of a wild predator (Mia Bisther, personal communication). To illustrate how dog attacks on livestock may contribute to societal conflict surrounding wolves, cases of dog attacks were included and compared alongside confirmed instances of wolf predation.

### Monthly indices of dog attacks

Using data from Rovbase and Swedish Media News, monthly indices of dog attacks on livestock were calculated: the average number of attacks per month was divided by the overall monthly average across all months:


$$I_{m}={\tilde{A}}_{m}/{\tilde{A}}_{\text{Total}}$$

I_m_ = Index value for month *m.*

Ã_m_ = Average number of attacks in month *m.*

Ã_Total_ = Overall average number of attacks per month across all months.

## Results

### Swedish news media

Google generated 752 media articles, of which 49 were included in the results. The Media Archive search generated 172 articles, of which 23 were included in the results. A search in Swedish Newspapers for ‘dog attack’ from 2000 to 2024 yielded 2,886 pages (articles) in 100 newspapers, and a search with combined keywords and exclusion of non-relevant articles and duplicates, 20 articles remained (Table 1).

**Table 1:** Article search from three news media sources.

	Google	Media archive	Swedish newspapers
**Search words in entire article**	dog attack AND pig OR swine OR cow OR horse OR sheep OR ewe OR lamb OR alpaca OR hen OR chicken OR goose OR duck		
**No of hits**	752	172	240
**Exclusion criteria**	dog attack on other than livestock, wrong time span, not Swedish dog attack, non-relevant		
**No of relevant articles**	130	44	34
**Exclusion criteria**	duplicate		
**No of relevant articles**	49	23	20
**Total no of articles**	92		

**Figure 1:**
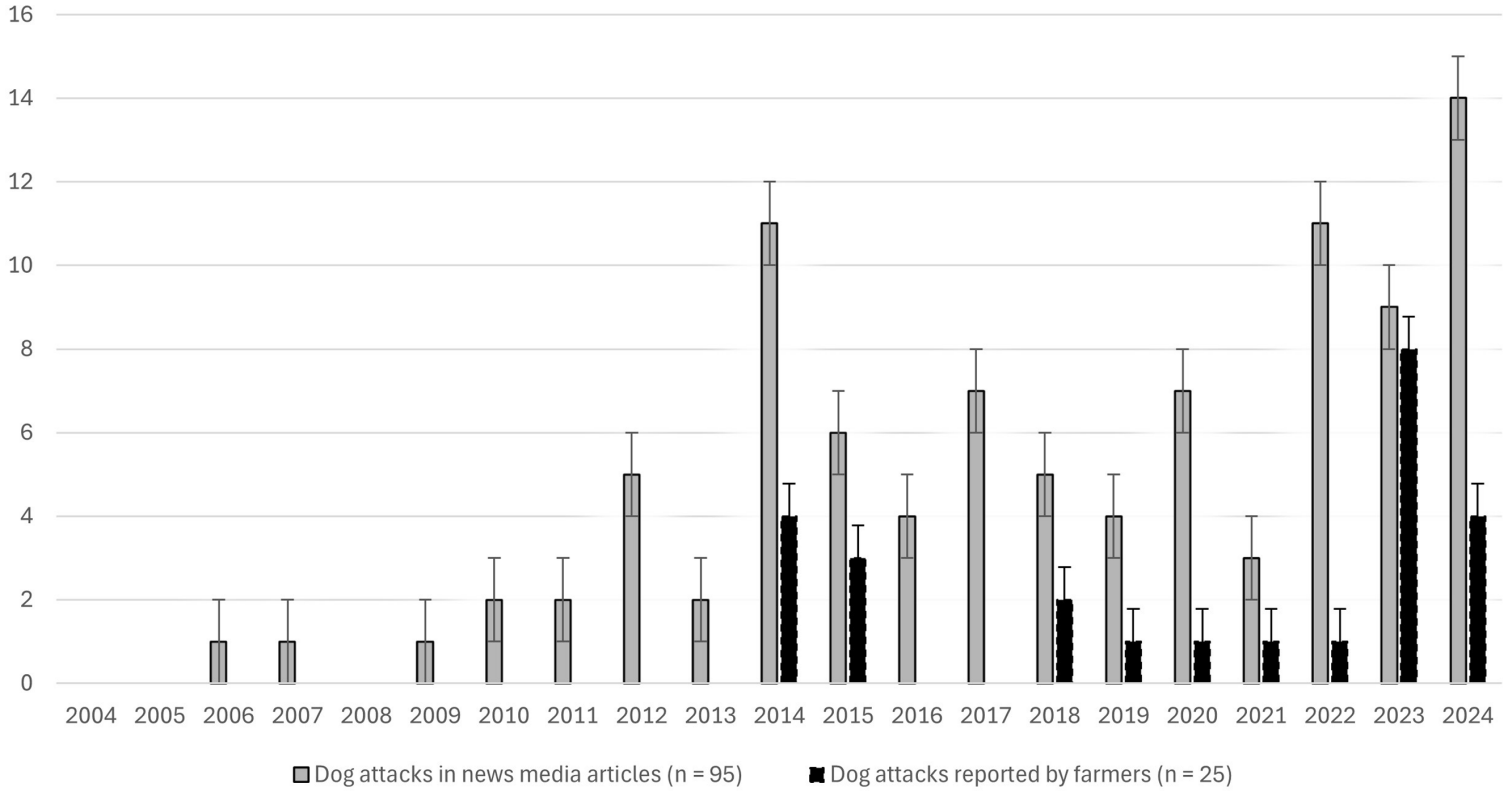
Dog attacks on livestock in Swedish news media articles 2006–2024 (*n =* 92) and dog attacks on livestock as reported by farmers (*n =* 25) in the survey from 2014 or earlier to 2024.

**Figure 2:**
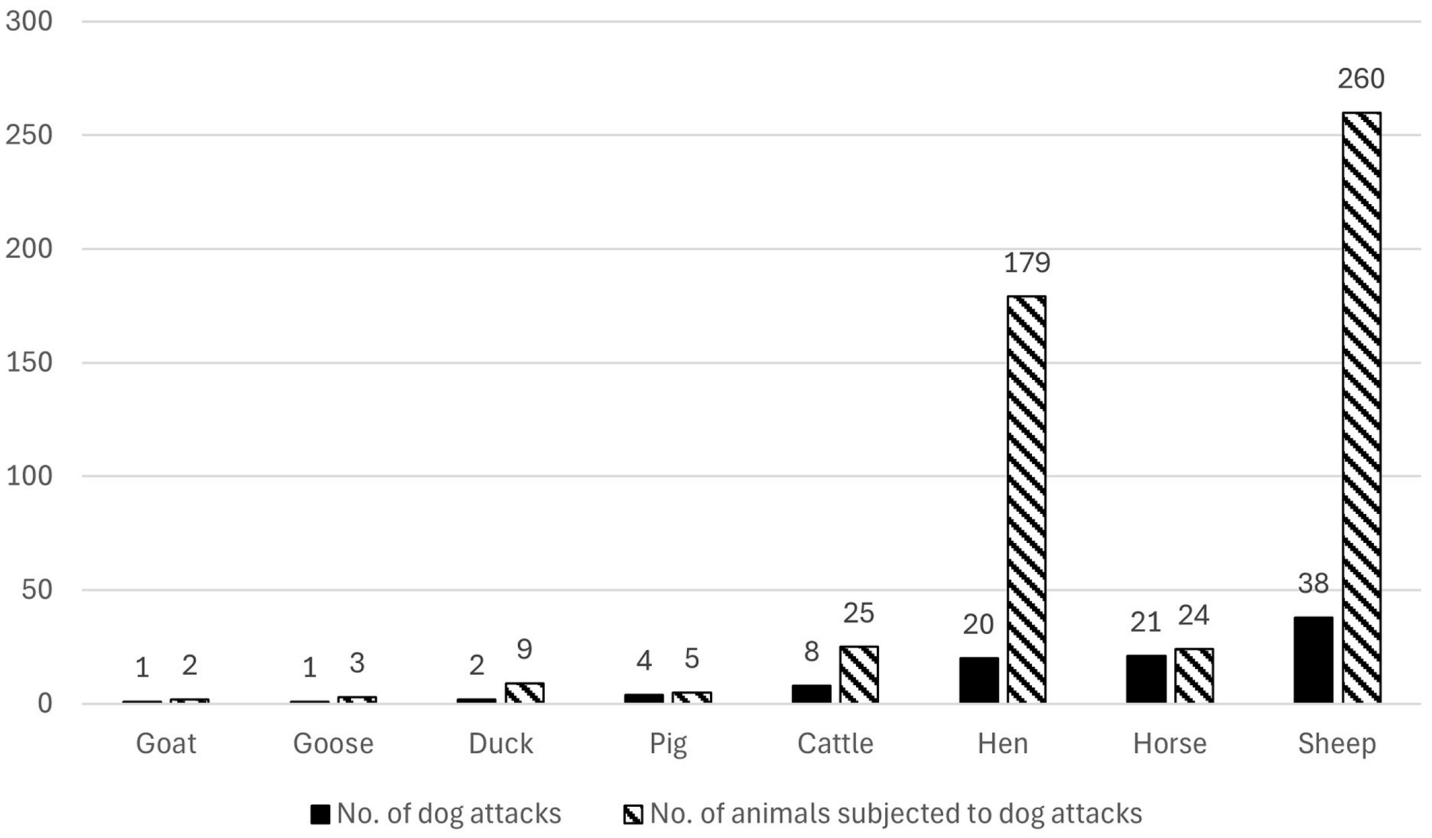
Number of dog attacks (*n =* 95) and number of injured or killed animals per livestock species (in total 507 animals) according to Swedish news media articles (*n =* 92) 2006–2024. One attack can involve several livestock species and several injured or killed animals.

Ninety-two news media articles about dog attacks on livestock were found in three Swedish news media sources, involving eight domestic animal species, totaling 507 animals (Figs. 1-2). Three articles reported that two different domestic animal species (cattle and pig; geese and duck; geese and hens) were subjected to a dog attack on the same occasion. The data from the news media articles shows a trend of increasing dog attacks over time from 2008, while survey answers from sheep farmers are mixed, with a noticeable spike in 2023 (Figure 1).

The livestock species most often described in the news media articles were sheep (*n =* 38), followed by horses (*n =* 21), and hens (*n =* 20), and the livestock species most frequently attacked by dogs were sheep, followed by hens and cattle (Figure 2).

**Figure 3:**
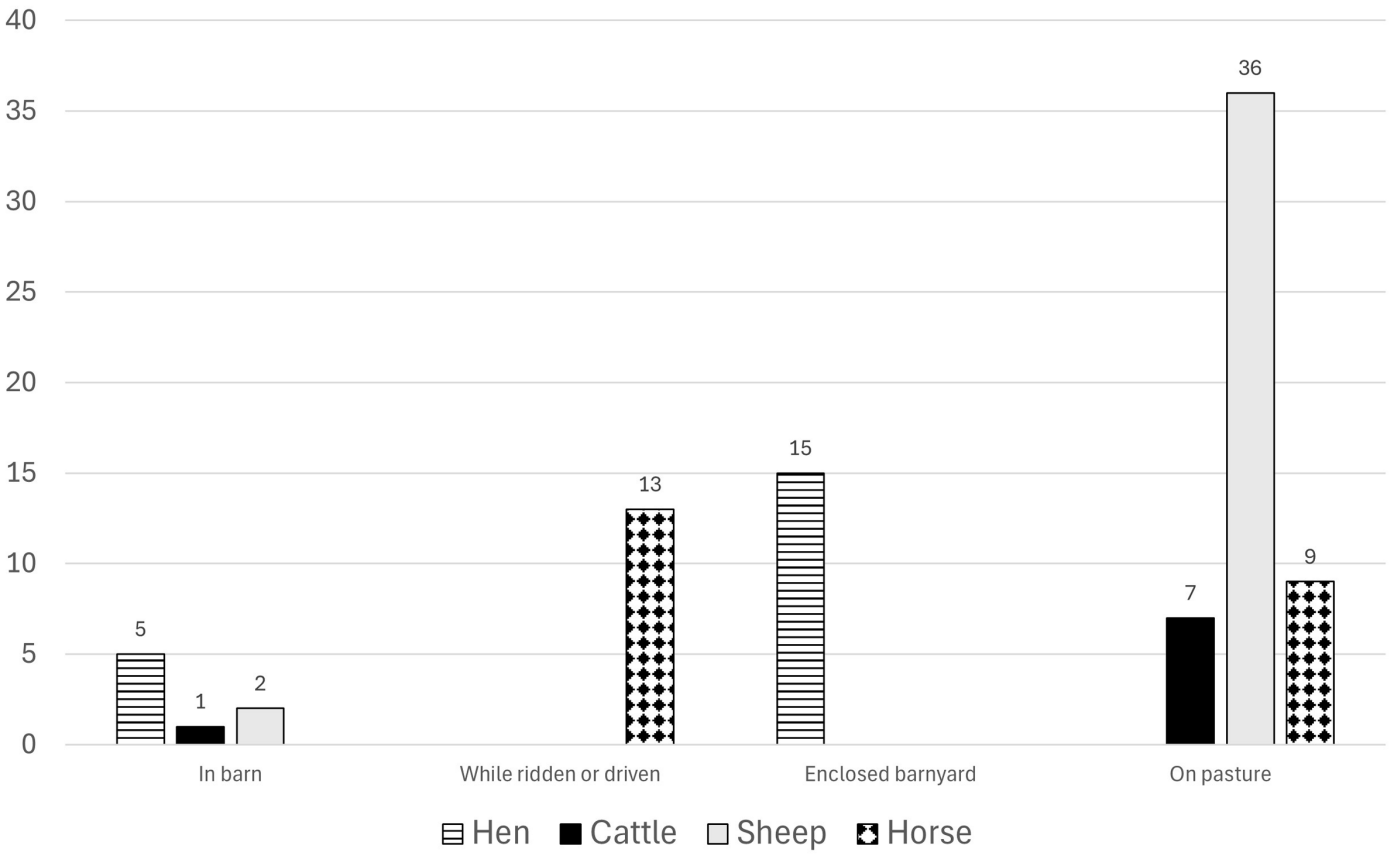
Circumstances during dog attacks on sheep, cattle, hen and horses according to Swedish news media articles (2006–2024).

The location of the dog attacks varied. Sheep were most often attacked in the pasture and hens in their enclosed barnyard or when they were loose. Horses (21 attacks, 23 animals) were most frequently attacked outdoors when ridden or driven (Figure 3). In four cases, the dogs also turned on the rider or driver during the attack, causing bite injuries to humans. In one case where the rider was saved by a passerby, the bite injuries on the rider were life-threatening.

The type and identity of 35% (*n =* 30) of the attacking dogs was known. Thirteen percent (*n =* 12) were of fighting dog type (i.e., “Bull type terrier,” according to the FCI nomenclature), e.g., American Staffordshire terrier “Amstaff,” Pitbull, American Bully, American Bulldog, Staffordshire Bull terrier, or the article identified the dog as a Bull type, e.g., Pitbull. Ten percent (*n =* 9) were of hunting type (e.g., Hound, Swedish Elkhound, Norwegian Elkhound, Drever, English Pointer, or the article identified the dog as hunting dog). Seven of the 12 Bull type terrier owners were present at the attack, while all the other dog owners were absent, regardless of the type of dog. Almost all dogs (*n =* 32) that were known to the livestock owner, besides the Bull type, were alone and loose when they attacked the animals. Also, a suspected wolf/dog hybrid, an Irish rescue dog, an Alaskan malamute and a herding-type dog were mentioned.

**Figure 4:**
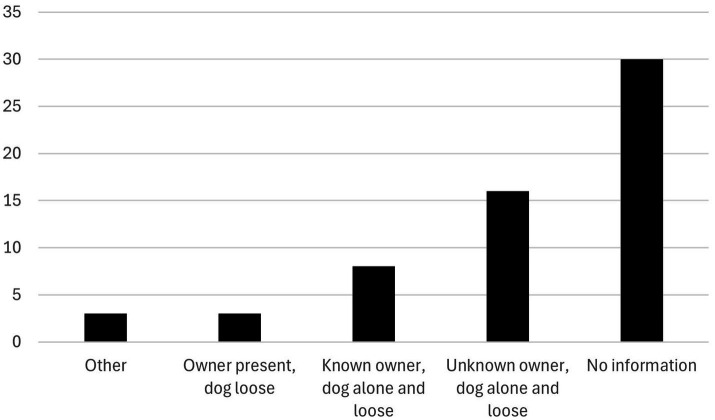
Attacks by unknown dogs (*n =* 60), including whether the owner was known and if the dog was alone and loose during the attack. Swedish news media articles 2006–2024.

Sixty-five percent of the attacking dogs (*n =* 60) were unidentified or unknown. In 30 of these, news media articles provided no information about the dog’s owner or whether the dog was alone or loose during the attack. In 16 cases, the articles did not specify the breed or type of the attacking dog but mentioned that the dog was loose and alone. In eight cases, the owner was known, but the dog responsible for the attack was not identified (Figure 4). News media articles indicated that livestock and horses frequently suffer not only from bite injuries, but also from being chased by dogs during dog attacks. For example, animals were sometimes driven through barriers, resulting in injuries also from their attempts to escape, such as running through fencing (not quantified in the study). In seven cases, dogs either broke free from the leash or escaped from their owners (e.g., from cars with open windows) and attacked the livestock with the owner present at the attack (Figure 4).

### Rovbase records from Västra Götaland 2001–2024

**Figure 5:**
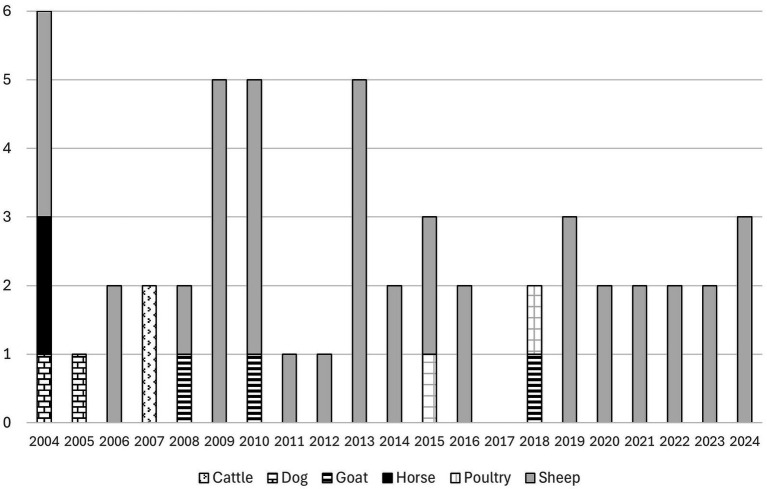
Livestock attacked by dogs. Identification was performed by the county administrative board inspectors of Västra Götaland and recorded in Rovbase 2004–2024 (*n =* 56). One attack on rabbits in 2015 is not included in the figure.

In total, 1,497 inspection records of predator attacks were documented by county administrative board inspectors between 2001 and 2024 in the Region Västra Götaland. Dogs accounted for 3.8% (*n =* 57) of all attacks that were inspected (Figure 5). A single attack could involve multiple domestic animal species, but the specific species detected concurrently were not specified in the report. Inspectors reported an average of 2.59 ± 1.62 dog attacks per year, with a median of two dog attacks (IQR 1).

**Figure 6:**
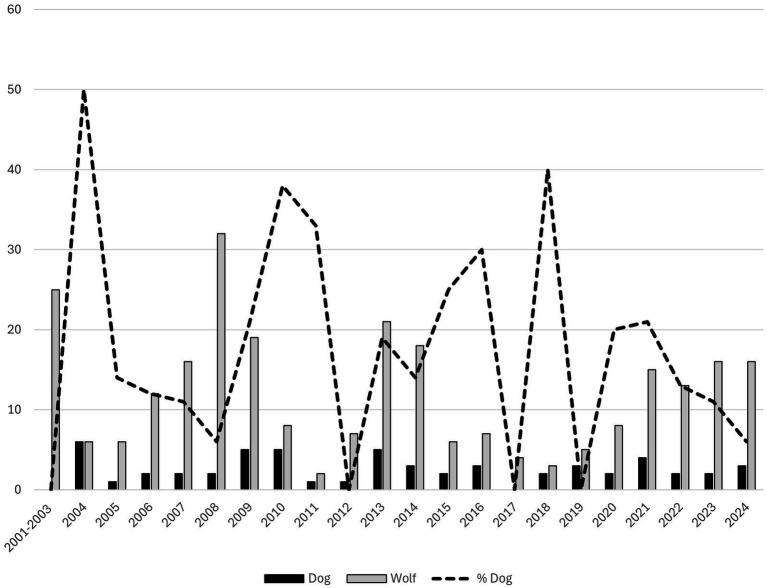
Number of wolf (*n =* 265) and dog attacks (*n =* 56) recorded to the Rovbase by County Administrative Board Inspectors (Västra Götaland, 2001–2024), along with the percentage of these attacks attributed to dogs, expressed as a share of the total (wolf and dog) attacks (dashed line).

The ratio between dog and wolf attacks on livestock reported in Rovbase varied substantially between years in Västra Götaland (range 0–50%, Figure 6). When wolf and dog attacks on livestock were combined, dog attacks accounted for an annual mean of 19.5% (SD = 13.5%), with a median of 16.5% (IQR 15.3), according to records from Rovbase in Västra Götaland.

### Time of the year for dog attacks

**Figure 7:**
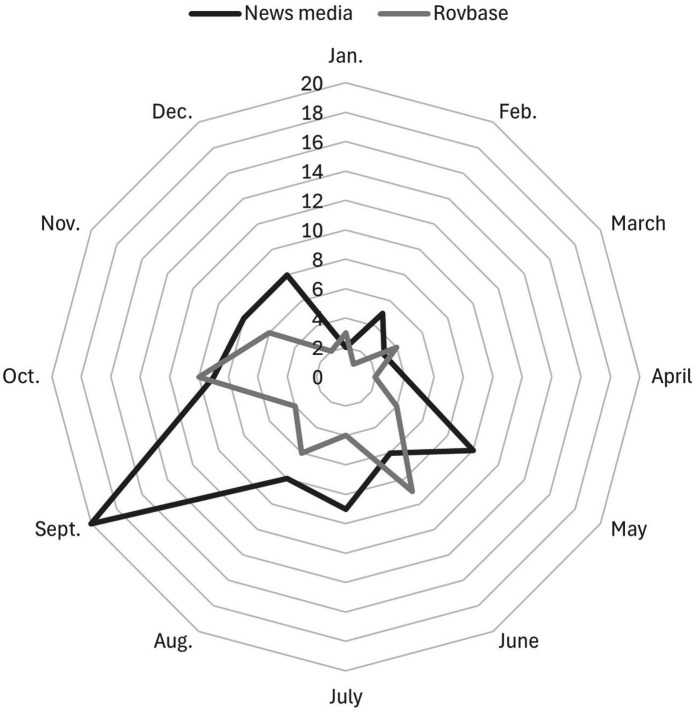
Time of the year dog attacks on livestock as reported in Swedish news media (*n =* 92) and in Rovbase, reported by county administrative board inspectors (*n =* 56).

Both the Swedish news media articles and the records of the county administrative board inspectors in Rovbase show that most dog attacks on livestock occur during the summer and fall months (Figure 7). Data from Rovbase indicate seasonal peaks in activity, with monthly indices (range 0.215–2.15) in June (1.92), August (1.33), October (2.15), and November (1.33), while the dog attacks in Swedish news media articles have a strong peak in September (2.60) and a weaker peaks in May (1.29), July (1.17) and October (1.17).

### Survey for inspectors

The survey of inspectors had a response rate of 47% (*n =* 56). The inspectors’ experience with inspections varied from having conducted 1–10 inspections (*n =* 15, 25%) to more than 200 inspections (*n =* 9, 16%), and 45% of the inspectors (*n =* 25) conducted between 11 and 50 inspections. The inspectors had experience in inspecting a wide range of animal species, including alpacas, bees (colonies), cats, cattle, hens, deer, dogs, ducks, horses and ponies, fallow deer, foxes, goats, mouflon sheep, rabbits, red deer, reindeer, sheep, and turkeys.

Sixty-four percent of the inspectors (*n =* 36) indicated that the livestock species most attacked by dogs was sheep. However, 20 percent of the inspectors (*n =* 11) omitted answering the question, most of them referring to a lack of experience. While 55% of the inspectors attributed 0–10% of their inspections to dog attacks, 9% reported that 10–20% were due to dog attacks and 36% claimed to not have inspected any dog attacks on livestock at all.

### Survey for farmers

Twenty-five farmers who had experienced dog attacks on their livestock responded to the survey. Of these, 76% (*n =* 19) were sheep farmers, 20% (*n =* 5) were alpaca farmers, and 4% (*n =* 1) were goat farmers. The herds subjected to dog attacks comprised between 2 and 175 individuals, with a mean population size of 19 ± 36.33 (SD). The total number of injured animals was 53, and the mean affected livestock by these dog attacks were 2.1 ± 1.59 (SD) animals with a median of 2 animals. Half of the farmers (*n =* 13, 52%) reported that animals were killed during the dog attack. In one case, 6–10 animals were killed; in the remaining fatal cases, between one and five animals were killed with a mean 1.03 ± 0.88 (SD).

Eighty-four percent of the attacking dogs (*n =* 21) was owned by a neighbor. Twelve percent of farmers (*n =* 3) reported that the dog owner was unknown. Most of the dogs reported by farmers in the survey as involved in attacks were identified as hunting dogs (52%; *n =* 13). Eighty-four percent of the farmers (*n =* 21) did not report dog attacks to any authority. Three farmers (12%) reported a dog attack on the county administrative board, and one farmer (4%) reported it to the police. Eighteen farmers (72%) in the survey expressed emotions such as anger, sadness, and concern for their animals and how heartbreaking it was to find a dead animal or a mutilated animal still fighting for its life.

## Discussion

### In what situations do dogs attack livestock and horses?

A prerequisite for dog attacks is that dogs are given the opportunity to gain access to animals. For example, a large part of broiler and pork production takes place in enclosed and highly controlled spaces where dogs do not have access. Therefore, it is often a small-scale environment where animals that are affected by dog attacks are kept outdoors, in addition to animals used for recreation. According to news media articles, sheep are most often attacked by dogs in pastures, hen outdoors in enclosed barnyards, or loose in yards in backyard systems. Pastures interspersed with trees or vegetative cover and flocks of sheep without supervision by either shepherds or guard dogs have been shown to be more vulnerable to predation (18), as could also be case in the incidents presented in this study.

One fifth of the responding farmers were alpaca keepers. Dog attacks on alpacas warrants more attention since the alpaca industry in Sweden is currently expanding; the number of alpaca facilities increased from 169 in December 2021 to 313 in July 2025, and the alpaca population grew from 2,113 to 3,313 during the same period (The Animal Registry Unit at the Swedish Board of Agriculture, personal communication, 2025). Young alpacas (crias) are considered particularly vulnerable to dog attacks, although adult alpacas may also suffer significant harm. In addition to bite-related injuries, alpacas may develop stress- or capture-induced myopathy as a result of such attacks, a condition that in severe cases can be fatal (29).

Attacks on horses are different as horses are more often attacked by dogs when ridden or driven than when grazing on pastures. In Sweden, 76% of all horses and 71% of locations housing horses were in or near urban areas in 2016 (30). Therefore, it is reasonable to assume that encounters between riders and dogs are common in woodlands and recreational areas near urban environments. Additional danger was that the riders were also kicked and trampled by the horses. Dogs escaping from cars or breaking free from the leash must be considered highly motivated and possibly different in behavior, and it could be argued that they actively seek opportunities to attack. In contrast, many livestock attacks may result from loose dogs that encounter livestock by chance, turning the situation into an opportunity for attack.

### What characteristics do dogs that attack have, and which animals are the targets of the attacks?

Among farmers, most of the dogs reported were hunting dogs, while in the media, hunting dogs were less reported. Hunting dogs have been mentioned as aggressors in earlier publications (8, 10). The true number of hunting dogs involved in attacks is likely underreported. In news media articles, most attacking dogs were unidentified, and no information was available for half of those cases. Given how many dogs were unidentifiable and lacked descriptive details, it’s probable that hunting dogs were involved more often than the data suggests. The affected livestock were found to be injured or dead without the animal caregiver seeing the attacking dog. However, signs of dog attacks were also evident. Dogs were identified in the remaining cases. Whether the dog owner was identified or known might have been kept out of the media, perhaps to maintain good neighbor relationships.

According to all four sources of this study, sheep were the most common animals killed or injured by dogs. The proportion of livestock animals killed by dogs, as reported by the different sources, ranged from 41 to 64%. This agrees with reports of dog attacks on livestock in other countries (31). Dogs are the primary cause of sheep and goat losses on small-scale livestock farms in Chile (32). Damage by predators to adult horses is quite uncommon compared to that in sheep (10, 33, 34). If rapid flight is not possible, horses can defend themselves against canid predators with foreleg strikes (35). In several media articles, the dog attacked not only the horse but also the horseback rider. This makes such incidents distinct, as both animals and humans were victims. Cattle and cows are known to attack dogs even if they do not have calves on their sides (36). Therefore, predation and injury in adult cattle are quite unusual. In the sheep farming sector, however, dog attacks may result in decreased productivity, underutilized pasture resources, and increased financial burdens for individual producers due to necessary investments in protective measures.

### Do the attacks occur by chance, or is there a discernible pattern – and are there similarities in similar studies from other countries?

Most dog attacks occur during the summer and fall months when livestock are grazing outdoors or in paddocks. They are usually performed by individual dogs, but dogs can cooperate in pairs or groups. Two dogs cooperated when killing four cattle during the same attack in Italy (19). A New Mexican sheep flock, consisting of 40 pregnant ewes, was also attacked by two dogs. During a dog attack, four ewes were killed, five later died due to complications from injuries, and over 75% of the remaining flock sustained injuries (37). In about 50% of the cases reported in Swedish news media articles, there was no information about attacking dogs. This suggests that livestock owners often find carcasses and injured animals after an attack. Many of these attacks might be opportunistic, as attacking dogs are often loose and unsupervised. In Sweden, it is against the law to have dogs loose during the summer months [Section 16 Swedish Act (2007:1150) on the supervision of dogs and cats]. The fact that many of the attacks occurred during these months is troublesome and illustrates a disregard for the law. It is also worth noting that none of the farmers seemed to have exercised their right to kill dogs that were harassing or injuring their animals. In Australia, it has been reported that domestic dogs were more often seen or heard of attacking livestock because they attack during the daytime (9). However, the witnesses did not destroy the attacking dog because of the absence of equipment, or because the dog was their own or belonged to a neighbor – most often living within a few hundred meters from the livestock being subjected to attack (9).

### What consequences did the attack have for the animals that were subjected to dog attacks?

According to Gregory (38), suffering associated with predation can be categorized into two distinct forms. First, there is acute distress resulting from pursuit and physical assault by predators. Second, chronic stress is induced by a persistent threat of predation. Furthermore, flocks that are subjected to regular predation exhibit heightened vigilance and become easily agitated, which can complicate their management during standard procedures (38). In our study, the animals were frightened, chased, injured, and killed by dogs. The bite wounds and subsequent infection caused suffering before they healed or until the animal died or was euthanized.

### Consequences for the livestock owners

The death or injury of livestock caused by dogs can negatively affect livestock production capacity, resulting in economic losses for the owner. If the inspection shows that damage to a domestic animal or dog is found to have been caused by a bear, lynx, wolf, golden eagle, or wolverine, the animal owner may receive compensation from the county administrative board. A trained inspector must be called if a large predator is suspected of having injured or killed a domestic animal or dog, even if the veterinarian has already examined the animal.

Dog attacks can be mistaken for wolf attacks (5, 7, 10, 39) and bites from large dogs can be easily confused with wolf bites (8), leading to inaccurate reporting, unjustly killing of wolves, worsen rural–urban tensions, increased public fear and incorrect compensation claims. It is also important to identify dogs that have attacked, injured, or even killed livestock in order to prevent further attacks by the same animal. Displaying hunting (or foraging) behavior is considered self-rewarding for a dog (40), and the risk of repeated attacks is high.

There are many reasons why dog attacks on livestock are underestimated. In Sweden, when a dog attacks livestock, the dog owner is solely legally responsible for the damage the dog causes to livestock. If the attacking dog is known, the dog owner and the livestock owner often settle amicably in private, and no record exists of the event. This might seem to be a great outcome, with no government involvement or ease of action. Maintaining a good relationship with one’s neighbors is very important, as bad relationships can have a great impact on day-to-day life (9, 41). Will agreement and compensation be fair, or will it better reflect the relationship between them? The most troublesome aspect is that if the livestock owner and dog owner cannot settle on compensation, then there is no available arbitration or other help for the parties. In this study, the alpaca and the sheep owners identified the neighbor as the owner of the attacking dog, and only three of the 25 attacks were due to unknown dogs. However, in the study’s media search, 50% of the dog attacks were caused by unknown dogs and owners. No rights or compensation are granted to the livestock owner for the loss of livestock. Elsewhere, livestock owners experienced helplessness when they were unable to locate the dogs responsible, and on the other hand also fear of retribution from the dog owner once a dog is destroyed (9). The livestock owners in our study felt emotional distress.

There is also a risk that dogs’ share of livestock predation is underestimated because of error bias in field observations conducted by local officials (5). This is not unexpected, considering that compensation for livestock losses is only granted when predation is due to wildlife carnivores and not dogs. False predation can be declared by livestock owners to obtain compensation (4). In a study by Mattiello et al. (21), only 35% of the predation incidents on sheep were verified as contributing to wolf predation, illustrating the difficulty in identifying the predating species and the risk of false attribution by the livestock owner. Perhaps part of the solution is to compensate for all canine predation, as some local administrations in Italy do to compensate for wolf predation, regardless of whether wolves, dogs, or wolf-dog hybrids were responsible for the damage (4). In Poland, livestock owners are compensated for livestock killed by wolves only and not livestock killed by dogs (7). Similarly, interviews with Finnish sheep farmers revealed that, surprisingly, dog owners were often not held accountable for the damage dogs caused, and the costs fell entirely on sheep owners (42), increasing the risk of conflict.

In Sweden, it is against the law to have dogs loose in nature from March 1 to August 20, but despite this, many of the dog attacks occurred during this time. The attacks mainly occurred from May to December, likely because of the increased outdoor activity of livestock, dogs, and their owners during this period. Rovbase records from inspectors do not necessarily end up in news media articles, and many articles during fall and late fall/early winter may reflect seasons for moose and deer hunting where hunting with dogs is permissible and could perhaps explain part of the spike in the number of dog attacks identified in the news media articles. It is not uncommon for hunting dogs to enter sheepfolds during big game hunting when the driven animal (e.g., an elk) runs through the sheep fence and tears it down. In this case, the dog can leave the elk as hunting prey and instead attack the sheep (8). Small lambs are usually bitten on their backs and shaken until death (8).

Nearly 50 percent of the inspectors reported that they had not inspected a livestock victim of a dog attack. Perhaps not surprisingly, many inspectors also stated a lack of experience. Considering the Rovbase data from Västra Götaland, where 3.8% of inspections were attributed to dog attacks, an inspector can perform many inspections without coming across a case in which a dog was the aggressor. However, our results indicate that dogs contribute negatively to the carnivore conflict in Sweden, as seen in statistics from Västra Götaland.

### Method limitations

Due to the lack of official statistics, we gathered data on dog attacks through Swedish news media articles and surveys of livestock owners and official inspectors. While news articles have been used elsewhere as data source when there is limited data (43), only a fraction of incidents are likely reported. More targeted search terms might also have yielded additional results.

Search engines like Google present challenges due to their proprietary and evolving algorithms, which prioritize established sources and may exclude smaller outlets (44). This limits replicability and can skew news searches. To address this, we also consulted local newspapers. The overlap between different news sources was substantial. Another limitation of the study was the very low response rate of the farmers. Perhaps the survey did not reach out to the farmers as expected, or for unknown reasons, only a few chose to answer. The response rate to a survey of Scottish sheep farmers was 21% (*n =* 9,148), which was considered high by researchers (45). In contrast to our study, Murray et al. (45) study used a mixed-methods approach that included surveys and interviews. We used a survey alone.

The survey of the inspectors had a higher response rate, suggesting that the topic was perceived as important and relevant by the target group.

The Rovbase records have certain limitations. The accuracy of carcass inspections conducted by board inspectors may be uncertain, and some dog attacks could be misattributed to other predators, or vice versa. However, assessing animal carcasses by inspectors can be challenging, especially if a long time has passed between the attack and inspection (10). Inspectors could benefit from additional training in recognizing signs of dog attacks and understanding the characteristic ways in which dogs handle prey. There are also differences in bite patterns depending on the size of the dog, and the anatomy of its skull (46).

## Conclusion

This study provides valuable insights on prevalence and nature of dog attacks on livestock in Sweden, from multiple perspectives: news media articles, farmers and regional authorities. Dog attacks appear opportunistic, often involving unsupervised, loose dogs targeting accessible livestock, primarily sheep, on pasture during summer and fall. Most attacks resulted in the animals being killed, often displaying a characteristic pattern of injuries. Dog attacks on equestrian units pose a significant threat to the life and health of both animals and humans.

Dog attacks on livestock can have serious societal consequences, such as being mistaken for wolf predation, and aggravating the inflamed human-wolf conflict. Dog owners have strict liability for the damage caused by the dog, but unidentified dogs and dog owners often leave livestock owners without financial compensation and with emotional distress. Preventive measures against dog attacks on livestock should be tailored to seasonal patterns and the specific target populations to ensure maximal effectiveness. Also, further research is warranted.

### Suggestions for the future

The consequences of dog attacks on livestock for farmers are both financial and emotional. Although official statistics and comprehensive data on the issue are lacking, the results presented here are sufficiently compelling to justify the proposal of targeted mitigation strategies. One could, for example, conduct information campaigns targeting specific groups such as alpaca farm visitors, horse owners and riders, about the risks associated with dogs and how to avoid dog attacks. Measures such as predator-deterrent fencing, currently used to prevent attacks by wild predators, also appear to be effective against dog attacks (47). However, alternative funding mechanisms are required, for instance, a dog tax allocated to a dedicated fund from which livestock owners may apply for financial support to install predator-deterrent fencing specifically aimed at preventing dog incursions on their properties. Nonetheless, systematic data collection and further research are needed to deepen our understanding of the problem’s full scope and to refine future interventions. Since dog owners are solely responsible for their animals, it may also be advisable to extend the period when dogs must be kept on a leash to cover the entire grazing season, when livestock are outdoors. This could be a viable legal measure, provided it is properly enforced. Finally, livestock owners should enhance the supervision of their animals during peak hunting seasons and engage in collaborative discussions with local hunting associations to develop appropriate mitigation strategies.

## Data Availability

The raw data supporting the conclusions of this article will be made available by the authors, without undue reservation.
